# Multiple Episodes of Cardiac Arrest Induced by Treatment With Ibogaine: A Case Report

**DOI:** 10.7759/cureus.63487

**Published:** 2024-06-29

**Authors:** David Mestre, Alexandra Paula, Francisco P Gil, José Vaz

**Affiliations:** 1 Intensive Care Unit, Hospital José Joaquim Fernandes, Beja, PRT

**Keywords:** polymorphic ventricular tachycardia, opioid dependence, cardiotoxicity, acquired long qt, torsade de pointes, cardiac arrest, ibogaine

## Abstract

Opioid dependence is a common problem, and therapeutic alternatives are scarce and ineffective. Ibogaine, illegal in several countries, has been reported as a possible therapy in alternative clinics and it is also used as a recreational drug, despite its cardiotoxic potential, including QT prolongation.

We report a case of long QT leading to multiple episodes of cardiac arrest after a single dose of ibogaine (200mg, 2.6mg/Kg) in a patient without structural heart disease. This case highlights the fact that even low doses of ibogaine can be lethal and warns us about the consequences of its use.

## Introduction

Opioid dependence is a condition associated with a high burden of disease and with a high relapse rate after treatment [1]. Ibogaine is a psychoactive alkaloid extracted from the root of *Tabernanthe iboga*, a shrub found in Africa. Ibogaine has been used in alternative clinics for the treatment of opioid dependence since it interacts with multiple neurotransmitters like N-methyl-D-aspartate (NMDA), κ- and μ-opioid receptors, and sigma-2 receptor sites [2]. Despite the potential effect as an anti-dependence drug, ibogaine can have effects on the cardiovascular system, including QT prolongation and arrhythmia [3].

Due to the concern about the safety of ibogaine and its toxicity, this drug was not approved by the Food and Drug Administration, and ibogaine use is illegal in several countries including the United States of America, Australia, Belgium, Denmark, France, Sweden, and Switzerland [4]. However, there are some countries with fewer legal restrictions where ibogaine is used for the treatment of opioid dependence in alternative Detoxification Centers or self-administered for recreational use [5]. We report a case of multiple episodes of cardiac arrest due to acquired long QT syndrome following the administration of ibogaine for opioid dependence.

## Case presentation

A 47-year-old man presented with a history of heroin dependence since he was 10 years old. He was on buprenorphine patches as an opioid withdrawal treatment, which he started six months before hospital admission. He was admitted to an alternative detoxification center to start ibogaine treatment three days before hospital admission. There was no other past medical history.

One hour after the pre-treatment test with 200 mg (2.6 mg/Kg, weight 76 Kg) of ibogaine at the detoxification center, the patient developed polymorphic ventricular tachycardia with cardiac arrest and was defibrillated by the local medical staff with the return of spontaneous circulation (ROSC) after one shock. The local emergency service was activated, and the patient was transferred to the hospital.

**Figure 1:**
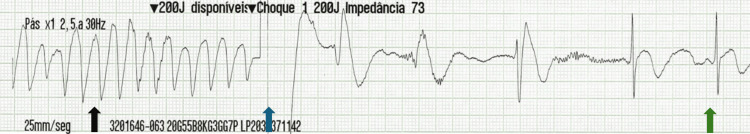
Electrocardiogram rhythm strip. Electrocardiogram (ECG) rhythm strip showing life-threatening arrythmia torsade de pointes (black arrow), defibrillation (blue arrow) and sinus rhythm (green arrow).

**Figure 2:**
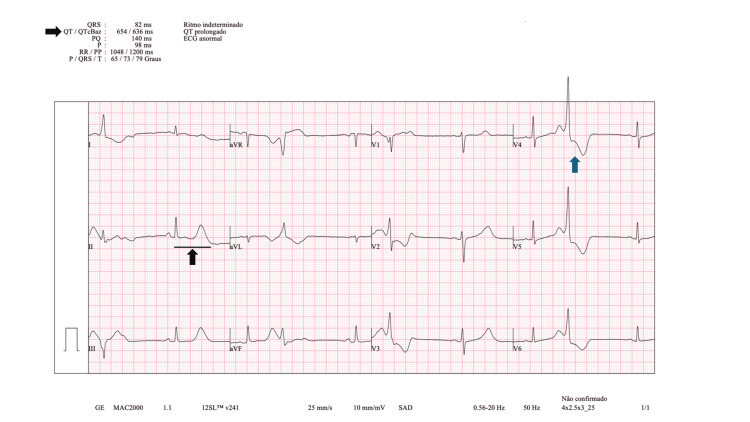
12-Lead electrocardiogram after ROSC. 12-Lead electrocardiogram (ECG) after return of spontaneous circulation (ROSC) showing QT prolongation (black arrows) - corrected (QTc) 636 ms (Bazett's formula) - and ventricular bigeminy (blue arrow).

After arrival at the emergency department, the patient developed two new episodes of polymorphic ventricular tachycardia with cardiac arrest (Figure 1); ROSC was observed after one shock in each episode. No neurological deficit was observed; the patient presented with spontaneous eye opening, oriented verbal response, and obeyed commands. Sinus bradycardia of 55 beats/minute was noted, and blood pressure and respiratory rate were normal. Arterial blood gas and laboratory analysis, including ions (sodium, magnesium, potassium and calcium), were normal. High-sensitivity cardiac Troponin T 1 hour after the first event was 4.9 ng/L (99th percentile upper reference limit: 14ng/L). Intoxication by other drugs (cocaine, benzodiazepines, alcohol, tricyclic antidepressants, barbiturates, and amphetamines) was excluded. The electrocardiogram (ECG) showed a corrected QT (QTc) interval of 636 ms (Bazett's formula was used) and ventricular bigeminy (Figure 2).

**Figure 3:**
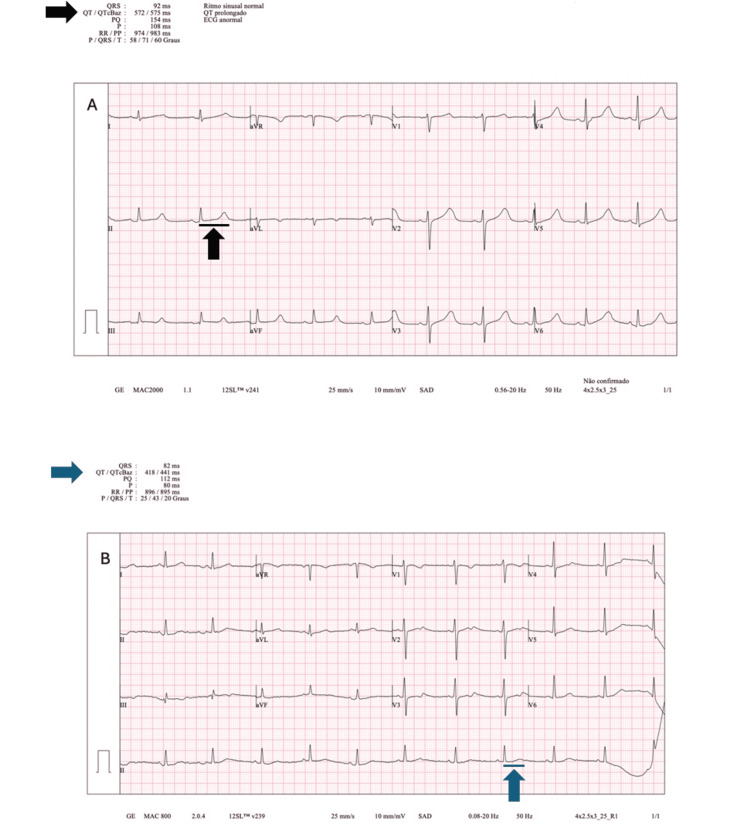
Serial 12-lead ECG exhibiting gradual QT normalization. A – Electrocardiogram (ECG) at day three with a corrected QT interval of 575 ms (black arrows). B – ECG at day eight with a corrected QT interval of 441 ms (blue arrows). Bazett's formula was used.

The patient was transferred to the Intensive Care Unit (ICU) for monitoring. In the ICU, the patient suffered another cardiac arrest after developing polymorphic tachycardia. One shock was administrated, and the patient presented ROSC. A lidocaine perfusion (2mg/minute) was started and maintained for 24 hours. Serial electrocardiograms were obtained with gradual improvement of QTc and normalization by day eight after the initial insult (Figure 3). The patient was discharged from the ICU and taken to the nursery on day nine.

Further investigation showed no abnormality, with a normal echocardiogram, treadmill stress test, coronary angiography, and cardiac magnetic resonance. No genetic test was performed to rule out congenital long QT because the patient was discharged against medical advice before collecting the sample.

Due to the absence of findings in the performed studies and QTc normalization, a long QT syndrome induced by ibogaine intoxication was diagnosed.

## Discussion

Opioid drug abuse has been rising among people in countries with higher incomes, and its dependence has been associated with an increase in mortality and morbidity [6]. Opioid dependence is a severe condition with limited therapeutic options, and the available therapies often have low efficacy. In the absence of an effective treatment with a low relapse rate, ibogaine became an alternative weapon for opioid dependence with promising outcomes [1].

Since the beginning of its clinical use, several doubts about ibogaine safety have been raised. Glicket al. [7] reported that high doses of ibogaine (100-200 mg/Kg) can decrease heart rate in mice without effects on blood pressure. In vitro studies show that ibogaine retards action potential repolarization of cardiomyocytes. Rubi et al. [8] concluded that ibogaine prolongs repolarization through human ether-a-go-go-related gene (hERG) potassium channel inhibition. Lamothe et al. [9] demonstrated that dysfunction of the hERG gene can cause long QT syndrome and sudden death. Knuijver et al. [10] performed a study in humans demonstrating an increase in corrected QTc after a single dose of ibogaine (10 mg/Kg) lasting more than 24 hours. No torsade de pointes were observed in this study. However, the relationship between QT prolongation and the risk of ventricular tachycardia and torsade de pointes is well-established in the literature [11].

Despite the concerns about ibogaine cardiotoxicity and its risks, this drug has been used primarily in two scenarios: without prescription or with prescription in some alternative centers in less regulated countries.

To our knowledge, there are rare cases of life-threatening arrhythmias related to ibogaine, probably underreported. Paling et al. [12] published three case reports of complications of ibogaine; one patient showed self-limited torsade de pointes on ECG, and the other two presented QT prolongation with sinus bradycardia. None of the patients suffered cardiac arrest, and the three of them presented mild hypokalemia. The reported dose of ibogaine was 3.5 g. Meisner et al. [13] reported a case of a patient found in asystole after an attempt of self-detoxication. The author reported an intake of 4 g of ibogaine. Steinberg et al. [14] presented a case of pulseless monomorphic ventricular tachycardia after intoxication with a single dose of 5.6 g of ibogaine. The author also reported QT prolongation associated with ventricular bigeminy in the post-defibrillation ECG. Hypokalemia acted as a co-trigger in this patient, according to the author.

Compared to the previously reported cases, the patient we reported also presented with a long QT after a single administration of ibogaine. Our patient required eight days to QTc return to normal, similar to the time reported by Steinberg et al. [14]. In our patient and in one previous case report, after defibrillation, the ECG showed ventricular bigeminy [14].

Differently from the other reported cases, our patient received a very inferior dose (200mg, 2.6mg/Kg), between 17.5 and 28 times inferior, showing that even a low dose of ibogaine can result in severe side effects. This fact brings us some new insights about Ibogaine toxicity and complications. The dose commonly used and thought of as safe according to the literature (8-25mg/Kg) [1] can be harmful and cause fatal arrhythmia. According to this data, ibogaine cardiotoxicity doesn’t appear to be strongly related to the administered dose. We also emphasize that in the case we report, there were no electrolyte deviations acting as confounding factors contributing to trigger ventricular arrhythmia.

The ibogaine intoxication case we describe, with multiple episodes of cardiac arrest, warns us of the need for continuous monitoring after ibogaine intake. QT alterations can last several days due to the long half-time of ibogaine. It also would be desirable to perform an ECG before treatment if this drug is considered.

Although knowledge of ibogaine is limited, the potential for related life-threatening complications is considerable. The use of ibogaine should be best regulated, and ibogaine prescribers should strongly consider the risks. 

## Conclusions

We report a rare cause of cardiac arrest related to ibogaine treatment. This case warns us about the severe risks of ibogaine, including a life-threatening side-effect. It is also important to alert healthcare providers about this drug and its complications since alternative therapists and drug users are still using ibogaine despite the concerns about its risk. Therefore, ibogaine use should be better regulated considering the consequences of its administration.
